# Comparison of real-world treatment outcomes of systemic immunomodulating therapy in atopic dermatitis patients with dark and light skin types

**DOI:** 10.1016/j.jdin.2022.09.006

**Published:** 2022-10-10

**Authors:** Angela L. Bosma, Wouter Ouwerkerk, Madeline J. Heidema, David Prieto-Merino, Michael R. Ardern-Jones, Paula Beattie, Sara J. Brown, John R. Ingram, Alan D. Irvine, Graham Ogg, Prakash Patel, Nick J. Reynolds, R.M. Ross Hearn, Mandy Wan, Richard B. Warren, Richard T. Woolf, Ariënna M. Hyseni, Louise A.A. Gerbens, Phyllis I. Spuls, Carsten Flohr, Maritza A. Middelkamp-Hup, TREAT NL registry and UK-Irish A-STAR Study Groups

**Affiliations:** a Department of Dermatology, Amsterdam UMC, Location Academic Medical Center, University of Amsterdam, Amsterdam Public Health Research Institute, Amsterdam Institute for Infection and Immunity, Amsterdam, the Netherlands; b NHRIS, National Heart Centre Singapore, Singapore; c Faculty of Medicine, Universidad de Alcalá, Madrid, Spain; d Unit for Population-Based Dermatology Research, St John's Institute of Dermatology, Guy's & St Thomas' NHS Foundation Trust and King’s College London, London, UK; e Clinical Experimental Sciences, Faculty of Medicine, University of Southampton, Southampton, UK; f Department of Dermatology, University Hospitals NHS Foundation Trust, Southampton, UK; g Department of Dermatology, Royal Hospital for Children NHS Trust, Glasgow, UK; h Centre for Genomic and Experimental Medicine, University of Edinburgh, Edinburgh, UK; i Department of Dermatology, Division of Infection & Immunity, Cardiff University, Cardiff, UK; j Department of Clinical Medicine, Trinity College Dublin, Dublin, Ireland; k MRC Human Immunology Unit, MRC Weatherall Institute of Molecular Medicine, University of Oxford, Oxford, UK; l Department of Dermatology, Institute of Cellular Medicine, Medical School, Newcastle University, Royal Victoria Infirmary and NIHR Newcastle Biomedical Research Centre Newcastle Hospitals NHS Foundation Trust, Newcastle upon Tyne, UK; m Department of Dermatology & Photobiology, Ninewells Hospital and Medical School, Dundee, UK; n Pharmacy Department, Evelina London Children's Hospital, Guy's and St Thomas' NHS Foundation Trust, London, UK; o Institute of Pharmaceutical Science, King's College London, London, UK; p Dermatology Centre, Salford Royal NHS Foundation Trust, NIHR Manchester Biomedical 17 Research Centre, University of Manchester, Manchester, UK; q St John’s Institute of Dermatology, Guy’s and St Thomas’ NHS Foundation Trust, London, UK

**Keywords:** atopic dermatitis, atopic eczema, ciclosporin, daily practice, dupilumab, effectiveness, ethnicity, methotrexate, morphology, race, registry, routine clinical care, safety, skin type, systemic treatment, AD, atopic dermatitis, AE, adverse event, DLQI, Dermatology Life Quality Index, DST, Dark Skin Type(s), EASI, Eczema Area and Severity Index, IQR, interquartile range, LST, Light Skin Type(s), NRS, Numerical Rating Scale, POEM, Patient-Oriented Eczema Measure, SD, Standard Deviation

## Abstract

**Background:**

Few data exist on differences in treatment effectiveness and safety in atopic dermatitis patients of different skin types.

**Objective:**

To investigate treatment outcomes of dupilumab, methotrexate, and ciclosporin, and morphological phenotypes in atopic dermatitis patients, stratified by Fitzpatrick skin type.

**Methods:**

In an observational prospective cohort study, pooling data from the Dutch TREAT (TREatment of ATopic eczema) NL (treatregister.nl) and UK-Irish A-STAR (Atopic eczema Systemic TherApy Register; astar-register.org) registries, data on morphological phenotypes and treatment outcomes were investigated.

**Results:**

A total of 235 patients were included (light skin types [LST]: Fitzpatrick skin type 1-3, *n* = 156 [Ethnicity, White: 94.2%]; dark skin types [DST]: skin type 4-6, *n* = 68 [Black African/Afro-Caribbean: 25%, South-Asian: 26.5%, and Hispanics: 0%]). DST were younger (19.5 vs 29.0 years; *P* < .001), more often had follicular eczema (22.1% vs 2.6%; *P* < .001), higher baseline Eczema Area and Severity Index (EASI) scores (20.1 vs 14.9; *P* = .009), less allergic contact dermatitis (30.9% vs 47.4%; *P* = .03), and less previous phototherapy use (39.7% vs 59.0%; *P* = .008). When comparing DST and LST corrected for covariates including baseline EASI, DST showed greater mean EASI reduction between baseline and 6 months with only dupilumab (16.7 vs 9.7; adjusted *P* = .032). No differences were found for adverse events for any treatments (*P* > .05).

**Limitations:**

Unblinded, non-randomized.

**Conclusion:**

Atopic dermatitis differs in several characteristics between LST and DST. Skin type may influence treatment effectiveness of dupilumab.

**Capsule Summary**

• There is a lack of knowledge about differences in treatment effectiveness and safety in atopic dermatitis patients of different skin types.
• Patients with atopic dermatitis differ in several characteristics between light and dark skin types, and skin type may influence treatment effectiveness of dupilumab in daily practice.

## Introduction

Atopic dermatitis (AD), also known as atopic eczema, is a chronic pruritic inflammatory skin disorder which is among the most common dermatological conditions. AD is more prevalent in black and mixed race populations, and differences seem to exist between AD in darkly pigmented and light skin, including variations in genetics and immunology.^1^, ^2^, ^3^, ^4^, ^5^, ^6^ Dark skin has been shown to have inherent structural properties that may trigger pruritus, such as higher transepidermal water loss and an increased size of mast cells.^7^^,^^8^ Higher natural moisturizing factor levels and down-regulated keratinocyte differentiation have been shown in dark skin compared to light skin, suggesting differences in pathophysiological mechanisms.^9^, ^10^, ^11^ This may imply a potential biological basis for differences in treatment response between light and dark skin. Clinically, AD can also present differently in a dark skin.^4^, ^5^, ^6^ Follicular eczema is an example of a morphological phenotype that is more frequently seen in African-American, Hispanic, and Asian patients.^12^ A systematic review confirmed differences in morphological AD characteristics by study region.^13^ Nevertheless, studies investigating the effectiveness and safety of systemic therapy in AD patients of different skin types are lacking, and only a few studies focus on this topic.^14^, ^15^, ^16^, ^17^, ^18^, ^19^, ^20^ Studies investigating treatments in AD patients are predominantly conducted on white patients.^14^

In this study we aimed to investigate the effectiveness and safety of dupilumab, ciclosporin, and methotrexate in AD patients with different skin types. In addition, we wanted to investigate the association between morphological phenotypes and skin types. We hypothesized that AD patients with dark skin types (DST) have different treatment outcomes and morphological phenotypes compared to patients with light skin types (LST). We specifically focused on skin type instead of ethnicity or race, as skin type could be determined more objectively. Ethnicity or race are complex terminologies that, in addition to skin color may also cover country of origin, physical features, cultural traditions, and the concept of mixed ethnicity. We hypothesized skin type to be a proxy for genetic differences between patients, underlying potential differences in pathophysiology, and subsequently, morphology and treatment responses.

## Methods

### Study design

We conducted a registry-embedded observational prospective cohort study, using real-world data from the Dutch TREAT (TREatment of ATopic eczema) NL (treatregister.nl) and UK-Irish A-STAR (Atopic eczema Systemic TherApy Register; astar-register.org) registries.

### Setting

Patients were included at 2 centers in the Netherlands (November 2017 to June 2020), and 13 centers in the United Kingdom (October 2018 to April 2021). Study visits were at baseline, 4 weeks, and then approximately every 3 months, alongside routine clinic appointments.

### Participants

Eligible patients were all children and adults with AD according to the U.K. working party’s diagnostic criteria, starting treatment with dupilumab, ciclosporin, and/or methotrexate in the context of routine clinical care. All dupilumab patients met the national criteria for dupilumab treatment, which stipulate prior treatment of at least 4 months with 1 or more conventional systemic therapies. Patients were allowed to use other systemic immunomodulating treatments and topical treatments concomitantly. The study size resulted from the inclusion of eligible patients in the abovementioned timeframes.

### Variables

Data collection was based on the TREAT Registry Taskforce core dataset.^21^^,^^22^ Data on Fitzpatrick skin type and morphological phenotype based on standardized proforma (eg, [non-]flexural eczema, palmar hyperlinearity, pompholyx, discoid eczema, nodular prurigo, follicular eczema, keratosis pilaris, erythroderma, and ichthyosis vulgaris; definitions included in Supplementary Material 1, available via Mendeley at https://doi.org/10.17632/s9y7fh7nbx.1) were collected. LST were defined as Fitzpatrick skin types 1 to 3 and DST as Fitzpatrick skin types 4 to 6. Effectiveness was analyzed using the Eczema Area and Severity Index (EASI),^23^ Numerical Rating Scale (NRS) peak pruritus past 24 hours,^24^ Patient-Oriented Eczema Measure,^25^ Dermatology Life Quality Index (DLQI), and Children's DLQI or Infants' Dermatitis Quality of Life Index.^26^ Safety was assessed through the reporting of adverse events at each visit (AEs, definitions are included in Supplementary Table IA, available via Mendeley at https://doi.org/10.17632/gfygbsw82y.1).

### Definition of treatment endpoint

In previous studies, comparison of the effectiveness of methotrexate and ciclosporin at the same predefined treatment endpoint was considered a disadvantage due to differences in speed of action.^27^^,^^28^ Therefore, we defined appropriate treatment endpoints per treatment. Methotrexate has a relatively slow onset of action, and we, therefore, chose 6 months as treatment endpoint. To allow direct comparisons, we chose the same endpoint for dupilumab, even though the drug has a faster onset of action. In our dataset, ciclosporin was often terminated before 6 months of treatment, for instance because of side effects or ineffectiveness. As ciclosporin has a fast onset of action, we therefore analyzed the data at 3 months instead.

### Statistical analyses

Patient characteristics, safety, and treatment discontinuation data were summarized using descriptive statistics and assessed during the entire follow-up period of this study. For univariate comparisons, Mann–Whitney tests and chi-squared tests were used as appropriate.

Baseline scores were compared to treatment endpoint scores using paired t-tests. To investigate differences between treatment groups in delta scores and the course of scores over time, we used linear mixed-effects models with an interaction between time and treatment. We modeled the change in scores over time, using natural cubic splines with the optimal degrees of freedom based on the minimal Bayesian Information Criterion. To test if there is a difference between skin types in scores during treatment, an ANOVA (Analysis of variance) test was conducted to assess the difference between the model with skin type and a model where this interaction term was removed. We included a random intercept for each patient and, in addition to skin type, included variables for which we found a significant difference between DST and LST in the models as potential confounders (including age, baseline severity score, follicular eczema, allergic contact dermatitis, and previous phototherapy use). Missing values for the covariates were included as unknown.

Effects were considered statistically significant if *P* < .05. Analyses were performed using SPSS 24.0 (IBM) and R version 3.4.1 (Foundation for Statistical Computing).

We have included a RECORD/STROBE checklist as Supplementary Material 2, available via Mendeley at https://doi.org/10.17632/6zrg834255.1.

## Results

### Baseline patient characteristics

In total, 235 patients were included (Table I). The majority of patients were male (59.1%), 67.7% were white, 156 patients (66.4%) had LST, and 68 patients (28.9%) had DST. Skin types of 11 patients were missing, and were excluded from analyses comparing skin types.

**Table I:** Baseline patient characteristics

	Study cohort (*n* = 235)^a^	Light skin type (*n* = 156, 66.4%)	Dark skin type (*n* = 68, 28.9%)	*P* value
Sex—no. (%): Male/Female	139 (59.1)/96 (40.9)	93 (59.6)/63 (40.4)	40 (58.8)/28 (41.2)	.91
Age, median (IQR)—years	26.0 (14.0-45.0)	29.0 (17.3-48.0)	19.5 (13.0-32.3)	**<.001**
Age of onset AD, median (IQR)—years^1^	0 (0-3)	0 (0-3)	0 (0-4)	.92
EASI, median (IQR)^2^	17.0 (9.175-27.325)	14.9 (7.6-25.8)	20.1 (10.8-30.6)	**.009**
NRS pruritus past 24h, median (IQR)^3^	7 (6-8)	7 (6-8)	7 (4-9)	.38
POEM, median (IQR)^4^	21 (16-24)	21 (16-24)	20 (13-24)	.67
DLQI, mean ± SD^5^	14.1 ± 7.0	13.8 ± 6.9	14.8 ± 7.2	.32
Patients per treatment group—no. (%)				
Dupilumab	168 (71.5)	121 (77.6)	42 (61.8)	
Methotrexate	65 (27.7)	37 (23.7)	22 (32.4)	
Ciclosporin	26 (11.1)	19 (12.2)	7 (10.3)	
BMI—median (IQR)^b^	24.7 (22.6-27.8)	24.7 (22.6-27.3)	24.8 (21.8-30.1)	.63
Educational status^c,6^				.28
ISCED 0-2: Early childhood, primary and lower secondary education	57 (24.3)	38 (24.4)	14 (20.6)	
ISCED 3-5: Upper secondary to short cycle tertiary education	103 (43.8)	73 (46.8)	27 (39.7)	
ISCED 6-8: Bachelor’s, Master’s, Doctoral or equivalent level	64 (27.2)	40 (25.6)	21 (30.8)	
Ethnicity—no. (%)^7^				**<.001**
White (Europe, Russia, Middle East, North Africa, USA, Canada, Australia)	159 (67.7)	147 (94.2)	4 (5.9)	
Black African, Afro-Caribbean	18 (7.7)	0 (0)	17 (25.0)	
Asian-Chinese	5 (2.1)	0 (0)	5 (7.4)	
South-Asian (India, Pakistan, Sri Lanka, Nepal, Bhutan, Bangladesh)	23 (9.8)	4 (2.6)	18 (26.5)	
Asian-other (Korea, China north of Huai River)	8 (3.4)	0 (0)	7 (10.3)	
Hispanic or Latino	1 (0.4)	1 (0.6)	0 (0)	
Mixed	19 (8.1)	4 (2.6)^d^	15 (22.0)^e^	
Other	1 (0.4)	0 (0)	1 (1.5)	
Fitzpatrick skin type—no. (%)^6^				**<.001**
I/II	17 (7.2)/87 (37.0)	17 (10.9)/87 (55.8)	0 (0)/0 (0)	
III/IV	52 (22.1)/29 (12.3)	52 (33.3)/0 (0)	0 (0)/29 (42.6)	
V/VI	29 (12.3)/10 (4.3)	0 (0)/0 (0)	29 (42.6)/10 (14.7)	
Fitzpatrick skin type—median (IQR)	3 (2-4)	2 (2-3)	5 (4-5)	**<.001**
Morphological phenotypes—no. (%)				
Flexural eczema^8^	169 (71.9)	113 (72.4)	49 (72.0)	.96
Non-flexural eczema^8^	173 (73.6)	116 (74.4)	51 (75.0)	.31
Palmar hyperlinearity^9^	64 (27.2)	45 (28.8)	18 (26.5)	.29
Pompholyx^10^	13 (5.5)	10 (6.4)	3 (4.4)	.84
Discoïd (syn. nummular) eczema^11^	7 (3.0)	4 (2.6)	3 (4.4)	.41
Prurigo nodularis^12^	14 (6.0)	6 (3.8)	7 (10.3)	.13
Follicular eczema^13^	19 (8.0)	4 (2.6)	15 (22.1)	**<.001**
Keratosis pilaris^14^	12 (5.1)	5 (3.2)	7 (10.3)	.09
Erythroderma^15^	14 (6.0)	9 (5.8)	3 (4.4)	.58
Ichthyosis vulgaris^16^	11 (4.7)	6 (3.8)	5 (7.4)	.34
Infraorbital Dennie-Morgan skin folds^17^	13 (9.8)	10 (10.5)	3 (7.9)	.53
Infra-auricular fissure(s)^18^	14 (10.5)	11 (11.6)	3 (7.9)	.29
Skin infection^19^	17 (7.2)	11 (7.1)	4 (5.9)	.95
Allergic co-morbidities—no. (%)				
Asthma^f,7^	128 (54.5)	87 (55.8)	41 (60.3)	.68
Allergic rhinoconjunctivitis^f,7^	129 (54.9)	92 (59.0)	37 (54.4)	.69
Atopic eye disease^f,20^	18 (7.7)	13 (8.3)	5 (7.4)	.53
Eosinophilic esophagitis^f,20^	2 (0.8)	1 (0.6)	1 (1.5)	.86
Allergic contact dermatitis^g^	97 (41.3)	74 (47.4)	21 (30.9)	**.026**
Food allergy	118 (50.2)^h^/93 (39.6)^i^	76 (48.7)^h^/63 (40.4)^i^	42 (61.8)^h^/30 (44.1)^i^	.18/.14
Family history of AD and allergic diseases^j,21^- no. (%)	140 (59.6)	98 (62.8)	42 (61.8)	.84
Previous use of systemic therapies for AD—no. (%)^5^	190 (80.9)	134 (85.9)	50 (73.5)	.052
Ciclosporin	127 (54.0)	89 (57.1)	35 (51.5)	.57
Azathioprine	38 (16.2)	29 (18.6)	6 (8.8)	.14
Methotrexate	96 (40.9)	64 (41.0)	28 (41.2)	.80
Mycophenolic acid/mycophenolate mofetil	30 (12.8)	19 (12.2)	10 (4.7)	.71
Systemic corticosteroids	99 (42.1)	76 (48.7)	23 (33.8)	.09
Dupilumab^k^	2 (0.9)	0 (0)	1 (1.5)	.26
Other medication^l^	2 (0.9)	2 (1.3)	0 (0)	.52
Investigational medication	14 (6.0)	11 (7.1)	3 (4.4)	.60
Previous use of phototherapy—no. (%)	122 (51.9)	92 (59.0)	27 (39.7)	**.008**
Concomitant immunomodulating therapy—no. (%)	37 (15.7)	24 (15.4)	13 (19.1)	.49
Systemic corticosteroids^m^/Other^n^	30 (12.8)/7 (3.0)	20 (12.8)/4 (2.6)	10 (14.7)/3 (4.4)	.70/.47

*AD*, Atopic dermatitis; *BMI*, body mass index; *DLQI*, Dermatology Life Quality Index; *EASI*, Eczema Area Severity Index; *IQR*, interquartile range; *ISCED*, International Standard Classification of Education; *No*., number; *NRS*, Numerical Rating Scale; *POEM*, Patient-Oriented Eczema Measure; *SD*, standard deviation.

Significant *P* values displayed in bold. Missing data: ^1^*n* = 15, ^2^*n* = 5, ^3^*n* = 33, ^4^*n* = 14, ^5^*n* = 16, ^6^*n* = 11, ^7^*n* = 1, ^8^*n* = 51-57, ^9^*n* = 25, ^10^*n* = 59, ^11^*n* = 62,^12^*n* = 59, ^13^*n* = 64, ^14^*n* = 22, ^15^*n* = 20, ^16^*n* = 58, ^17^*n* = 53, analysis of NL data, ^18^*n* = 56, analysis of NL data, ^19^*n* = 16, ^20^*n* = 2, ^21^*n* = 10.

^a^AD based on the U.K. Working Party’s Diagnostic Criteria: *n* = 133 (NL), *n* = 102 (UK), ^b^Excluding patients <18 years, ^c^<18 years: ISCED of parents, ^f^physician-diagnosed, ^g^positive patch test; never tested (*n* = 24), tested negative (*n* = 15), unknown (*n* = 12) or missing (*n* = 87), ^h^patient-reported, ^i^patient-reported food allergy was confirmed by a physician diagnosis; patient-reported food allergy (*n* = 131), ^j^first degree family member with at least one of the following allergic diseases: AD, asthma, allergic rhinoconjunctivitis, atopic eye disease or other, ^k^open-label extension study, ^l^dimethyl fumarate (*n* = 1), rituximab (*n* = 1), ^m^predniso(lo)ne, ^n^ciclosporin (*n* = 3), long-term clarithromycin (*n* = 1), methotrexate (*n* = 1), mycophenolate mofetil (*n* = 1), ciclosporin and dupilumab concomitantly (*n* = 1).

DSTs were on average younger when entering the registries compared to LSTs (median age 19.5 vs 29.0 years; *P* < .001). Higher baseline EASI scores were recorded in DST (20.1 vs 14.9; *P* = .009). Allergic contact dermatitis and previous use of phototherapy were more prevalent in LST (47.4% vs 30.9%; *P* = .026 and 59.0% vs 39.7%; *P* = .008, respectively). We also found a correlation between ethnicity and skin type (*P* < .001).

### Effectiveness according to skin type

In total, 168 patients were treated with dupilumab (LST: *n* = 121 [72.0%], DST: *n* = 42 [25.0%]), 65 patients with methotrexate (LST: *n* = 37 [56.9%], DST: *n* = 22 [33.8%]), and 26 patients with ciclosporin (LST: *n* = 19 [73.1%], DST: *n* = 7 [26.9%]).

**Fig 1:**
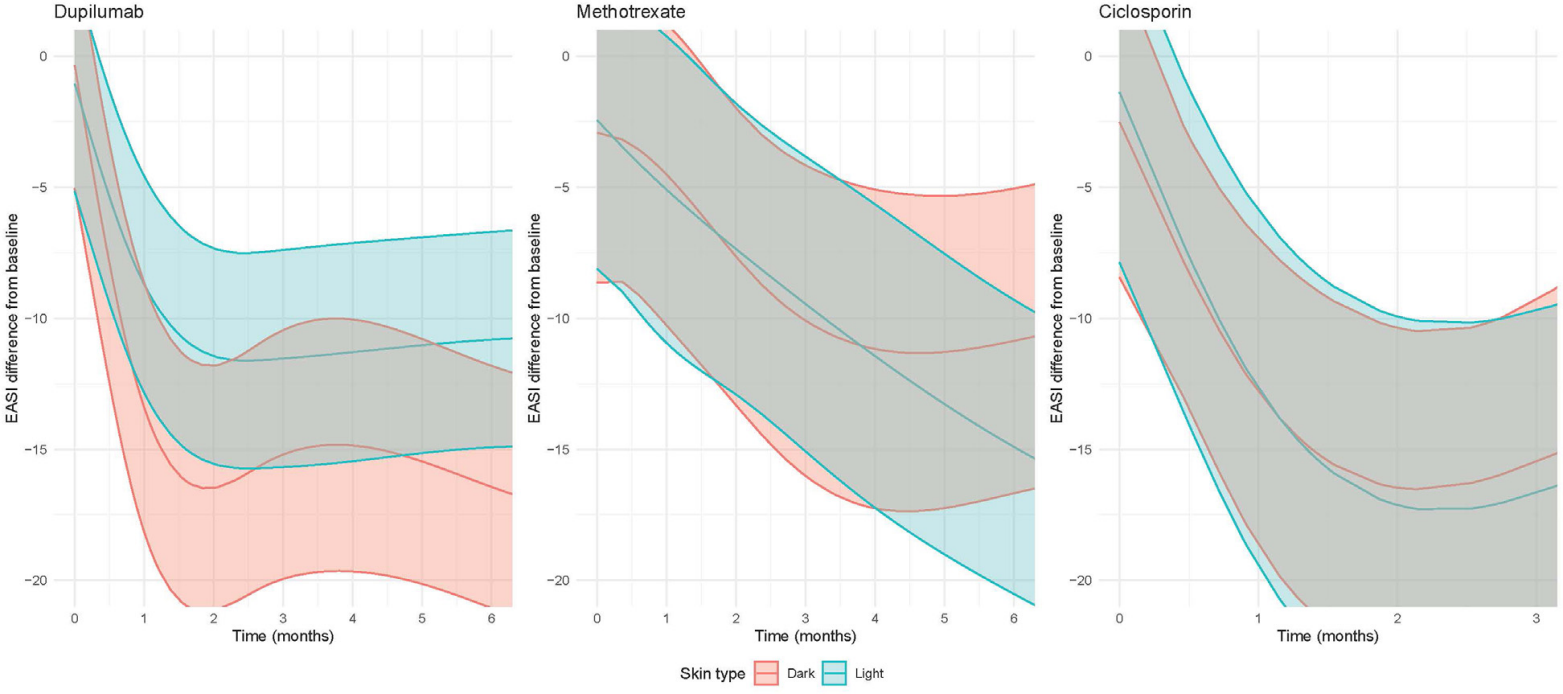
Difference in Eczema Area and Severity Index (EASI) from baseline (delta EASI) over time for each treatment group. Estimated mean differences in EASI scores from baseline (including 95% confidence interval) for our linear mixed-effects models with continuous values for time, and time displayed in weeks and corrected for age, baseline EASI scores, follicular eczema, allergic contact dermatitis, and previous use of phototherapy, in patients with atopic dermatitis. Higher delta scores indicate greater improvement of disease activity and/or burden. The median follow-up duration for the outcome measurements varied from 38 to 46 weeks (IQR: 14-74 weeks) for dupilumab, from 17 to 19 weeks (IQR: 1-47 weeks) for methotrexate, and from 15 to 17 weeks (IQR: 0-32 weeks) for ciclosporin. Dupilumab: *n* = 168 at baseline (light skin types [LST]: *n* = 121; *dark skin* types [DST]: *n* = 42), *n* = 125 at 6 months (LST: *n* = 90; DST: *n* = 35). Methotrexate: *n* = 65 at baseline (LST: *n* = 37; DST: *n* = 22), *n* = 25 at 6 months (LST: *n* = 15; DST: *n* = 10). Ciclosporin: *n* = 26 at baseline (LST: *n* = 19; DST: *n* = 7), *n* = 15 at 3 months (LST: *n* = 11; DST: *n* = 4). *EASI*, Eczema Area and Severity Index.

For dupilumab and methotrexate, an ANOVA test revealed a significant *P* value for skin type as interaction term for EASI (*P* < .001 and *P* = .04, respectively), indicating that the course of EASI over time differs between DST and LST. Results of the linear mixed-effects models displaying the course of the scores over time according to skin type are shown for EASI only (Fig 1). Both skin type groups show improvement over time. Other scores are shown in Supplementary Figs 1-3, available via Mendeley at https://doi.org/10.17632/4khfjgm2n7.1, https://doi.org/10.17632/hscd3ysgvd.1, https://doi.org/10.17632/dcxtdbg86k.1.

To get insight into how DST and LST are different, we compared baseline scores to treatment endpoint scores (Table II). Significant improvement over time was observed for all outcome measures in both skin type groups when treated with dupilumab (eg ΔEASI for DST: 16.7; *P* < .001 and ΔEASI for LST: 9.7; *P* < .001). LST also showed significant improvements in all outcome measures for methotrexate (eg ΔEASI: 11.0; *P* = .019) and ciclosporin (eg ΔEASI: 13.1; *P* < .001). In DSTs treated with methotrexate and ciclosporin, EASI showed significant improvements for methotrexate (Δ5.7; *P* = .048) and borderline significant improvements were found for DLQI (Δ4.9; *P* = .051) for methotrexate, and EASI for ciclosporin (Δ12.9; *P* = .054). Both groups reached the minimal clinically important difference (MCID)^29^, ^30^, ^31^ for all outcomes with dupilumab. For methotrexate, patients with DST did not reach the MCID for EASI, Patient-Oriented Eczema Measure, and NRS pruritus. For ciclosporin, DST did not reach the MCID for NRS pruritus. When comparing DST and LST, DST showed a significantly greater improvement in EASI when treated with dupilumab, even after adjustment for age, baseline severity, follicular eczema, allergic contact dermatitis, and previous phototherapy use (Δ16.7 vs Δ9.7; *P* = .032; Table II). We found no difference in EASI improvement between DST and LST for methotrexate and ciclosporin, as well as no difference in any of the other scores for all treatments.

**Table II:** Effectiveness of dupilumab, methotrexate, and ciclosporin according to skin type

	Baseline score	Follow-up score	*P* value†	Δ score
Dupilumab				
EASI				
Mean score dark skin type (SD)	24.2 (13.0)	7.5 (7.1)	**<.001**	16.7 (13.0)
Mean score light skin type (SD)	18.0 (13.0)	8.3 (7.5)	**<.001**	9.7 (11.0)
*P*-value Δ difference‡				**.032**
POEM				
Mean score dark skin type (SD)	20.2 (6.0)	10.1 (6.0)	**<.001**	10.1 (6.4)
Mean score light skin type (SD)	19.9 (5.7)	10.5 (6.8)	**<.001**	9.4 (6.8)
*P*-value Δ difference‡				.33
DLQI				
Mean score dark skin type (SD)	15.6 (6.8)	6.2 (7.6)	**<.001**	9.4 (8.5)
Mean score light skin type (SD)	14.1 (6.7)	5.7 (5.7)	**<.001**	8.4 (7.3)
*P*-value Δ difference‡				.54
NRS				
Mean score dark skin type (SD)	6.9 (1.8)	3.5 (2.2)	**<.001**	3.4 (2.3)
Mean score light skin type (SD)	7.2 (2.3)	3.4 (2.7)	**<.001**	3.7 (3.0)
*P*-value Δ difference‡				.99
Methotrexate				
EASI				
Mean score dark skin type (SD)	12.9 (9.2)	7.2 (3.9)	**.048**	5.7 (7.4)
Mean score light skin type (SD)	19.0 (13.2)	7.9 (5.8)	**.019**	11.0 (14.7)
*P*-value Δ difference‡				.52
POEM				
Mean score dark skin type (SD)	13.8 (9.5)	10.5 (7.8)	.32	3.2 (8.5)
Mean score light skin type (SD)	18.5 (9.6)	10.9 (6.8)	**.007**	7.5 (8.4)
*P*-value Δ difference‡				.19
DLQI				
Mean score dark skin type (SD)	9.9 (6.9)	5.0 (3.5)	.051∗	4.9 (5.9)
Mean score light skin type (SD)	12.6 (8.3)	7.0 (7.5)	**.011**	5.6 (6.0)
*P*-value Δ difference‡				.26
NRS				
Mean score dark skin type (SD)	5.2 (3.1)	3.8 (2.3)	.17	1.3 (2.1)
Mean score light skin type (SD)	5.9 (2.9)	3.2 (2.2)	**.037**	2.7 (3.2)
*P*-value Δ difference‡				.74
Ciclosporin				
EASI				
Mean score dark skin type (SD)	23.2 (14.7)	10.3 (14.4)	.054∗	12.9 (8.3)
Mean score light skin type (SD)	21.3 (8.5)	8.2 (11.4)	**<.001**	13.1 (6.9)
*P*-value Δ difference‡				.98
POEM				
Mean score dark skin type (SD)	19.8 (9.3)	13.5 (8.4)	.29	6.2 (9.7)
Mean score light skin type (SD)	19.6 (6.4)	8.0 (9.3)	**.008**	11.6 (9.9)
*P*-value Δ difference‡				.39
DLQI				
Mean score dark skin type (SD)	16.2 (9.0)	6.8 (7.3)	.12	9.5 (8.7)
Mean score light skin type (SD)	13.8 (5.7)	3.1 (2.4)	**<.001**	10.7 (5.9)
*P*-value Δ difference‡				.36
NRS				
Mean score dark skin type (SD)	7.2 (2.2)	5.0 (2.9)	.25	2.2 (3.2)
Mean score light skin type (SD)	7.1 (2.2)	2.4 (2.5)	**.005**	4.7 (3.7)
*P*-value Δ difference‡				.63

Mean scores (SD) for dark and light skin type at baseline and follow-up (6 months dupilumab, 6 months methotrexate, 3 months ciclosporin), and the corresponding differences for each skin type. Δ-score: reduction in score between baseline and follow-up.

Significant *P* values displayed in bold.

The minimal clinically important difference for improvement is a decrease of 6.6 points for EASI, 3.4 points for POEM, 3.3 points for DLQI, and 2.7 points for NRS pruritus.

*DLQI*, Dermatology Life Quality Index (0-30); *EASI*, Eczema Area and Severity Index (0-72); *NRS*, Numerical Rating Scale (0-10); *POEM*, Patient-Oriented Eczema Measure (0-28).

∗ Borderline significant.

† Paired t-tests for comparison between baseline and follow-up. Number of patients per treatment group: dupilumab: dark: *n* = 35, light: *n* = 90; methotrexate: dark: *n* = 10, light: *n* = 15; ciclosporin: dark: *n* = 4, light: *n* = 11.

‡ The *P*-value Δ difference between the Δ-scores for light and dark skin type was assessed according to a multivariable linear model, corrected for age, baseline score, follicular eczema, allergic contact dermatitis and previous use of phototherapy.

### Concomitant therapy during follow-up

In total, 31 (18%), 13 (20%), and 7 (27%) patients used conventional systemic therapy concomitantly with dupilumab, methotrexate, and ciclosporin, respectively (Supplementary Table IIA-C, available via Mendeley at https://doi.org/10.17632/9v46888y3v.1). No differences were found for usage of concomitant systemic therapy or mean usage duration between DST and LST (*P* > .05).

### Safety

In total, 79 potentially related adverse events were reported during the study (Supplementary Table IA-C). No serious adverse events were reported. In none of the treatment groups, differences were found in the total number of adverse events when comparing DST and LST (*P* > .05).

### Treatment discontinuation

A significant difference in treatment discontinuation was found between treatments, with most discontinuation for ciclosporin (*n* = 12/26, 46.2%), followed by methotrexate (*n* = 20/65, 30.8%), and dupilumab (*n* = 23/168, 13.7%) (*P* < .001). The most frequent reasons for discontinuation were side-effects and/or treatment ineffectiveness (Supplementary Table III, available via Mendeley at https://doi.org/10.17632/kfz4sb8sbt.1). However, no differences in treatment discontinuation were found between DST and LST (*P* > .05).

### Differences in morphological phenotypes

We found a higher prevalence of follicular eczema in DST (22.1% vs 2.6%; *P* < .001) (Table I). No differences were found between skin types for the other morphological features ([non-]flexural eczema, palmar hyperlinearity, pompholyx, discoid eczema, nodular prurigo, keratosis pilaris, erythroderma, ichthyosis vulgaris, infraorbital Dennie–Morgan skin folds, and infra-auricular fissure[s]). No analyses could be performed to investigate, if the morphological phenotypes respond differently to treatment due to low numbers.

## Discussion

In this study we investigated treatment outcomes and morphological phenotypes in AD patients with DST versus LST receiving treatment with dupilumab, methotrexate, and ciclosporin in a daily practice setting. Patients with DST had significantly more severe disease at baseline, indicated by higher EASI. We found that EASI scores improved in both DST and LST when treated with dupilumab, methotrexate, and ciclosporin, although this change did not reach statistical significance in DST ciclosporin patients, probably related to the small sample size. When comparing treatment effectiveness between DST and LST, DST patients showed a significantly greater EASI improvement in comparison to LST when treated with dupilumab after correction for baseline differences, but not with methotrexate or ciclosporin. No differences were found between DST and LST for total number of adverse events. Taken together, skin type may potentially influence treatment effectiveness of dupilumab, but does not seem to affect safety. Concerning morphological phenotypes, follicular eczema was significantly more common in DST.

DST patients had significantly higher baseline EASI scores, indicating more severe disease at the time of inclusion. DSTs were also significantly younger. Higher disease severity in DST has been reported previously,^1^, ^2^, ^3^, ^4^ and a retrospective study showed that children with treatment resistant AD more often had DST.^32^ Patients with skin type IV were also found to have higher scores of EASI, DLQI, and Investigator Global Assessment, compared to patients with Fitzpatrick skin type II.^33^ Nonetheless, our registries contain more patients with LST than DST. This may result from the geographical location of the including centers, or it may reflect a potential disparity in receiving systemic therapies amongst the subgroups. Other studies showed racial and ethnic disparities in receiving therapies for AD and other diseases.^34^^,^^35^ Black psoriasis patients are reported to be less likely to receive biologics than white patients due to potential financial and racial barriers in the United States.^36^ More research on disparities in receiving systemic AD therapies and potential causal factors of differences in severity amongst subgroups would be of interest.^37^, ^38^, ^39^

We found that allergic contact dermatitis was more prevalent in LST versus DST. Dark skin has been shown to be less *p*ermeable compared to light skin,^40^^,^^41^ and this could be a possible explanation. Another explanation could be that allergic contact dermatitis is more difficult to diagnose in DST. However, it may also be possible that LST are more commonly investigated for contact allergy, for example because they have better access to health care. The higher numbers of previous phototherapy in LST could be explained by a higher age in this subgroup. No statistically significant differences were found between DST versus LST for other characteristics, such as age of onset, body mass index, educational status, family AD history and allergic diseases, and concomitant therapy use.

Regarding morphology, we found significantly more follicular eczema in DST. Others have described follicular eczema in Hispanic and Asian populations,^12^^,^^42^, ^43^, ^44^ rather than directly comparing populations or focusing on skin type as was done in this study. Follicular eczema is characterized by follicular prominence clinically and follicular spongiosis histopathologically.^12^ Remarkably, the investigated morphological characteristics (eg pompholyx, discoid eczema, nodular prurigo, keratosis pilaris, erythroderma, and ichthyosis vulgaris) were only present in a small minority of patients. Due to limited numbers, we were not able to investigate treatment effects within morphological phenotypes.

In our registries, dupilumab was most frequently prescribed (71%), followed by methotrexate (28%), and ciclosporin (11%). Interestingly, prescription of methotrexate was more common than ciclosporin, despite the latter being an on-label treatment option for adults. For all treatments, side-effects were the main reason for discontinuation of treatment, followed by ineffectiveness.

Several limitations result from the daily practice setting. Due to the absence of randomization for treatment allocation, differences may arise in treatment groups because of selection bias. Dupilumab treatment requires previous use of conventional systemics. Also, bias may have been induced by the non-blinded observational nature of the study, including for severity assessments, with erythema being particularly difficult to assess in DST. We also had relatively low numbers of DST, especially in the methotrexate and ciclosporin groups. We did not stratify patients based on treatment dosage and included patients on combined systemic therapies. Only severe AEs were registered in the Netherlands as part of the TREAT core dataset.^22^

In summary, we found significant differences between AD patients with DST and LST, such as more severe disease at baseline and more follicular eczema in DST. Importantly, skin type may also influence treatment effectiveness of dupilumab in AD, as DST showed significantly greater EASI improvement than LST. Larger studies are needed to confirm these results, and skin type should therefore be considered a confounder in future AD intervention studies. Moreover, further research investigating whether morphological phenotypes respond differently to treatments is needed.

## Conflicts of interest

A. L. Bosma, S. J. Brown, P. I. Spuls, C. Flohr, and M. A. Middelkamp-Hup are investigators on the European Union Horizon 2020-funded BIOMAP Consortium (http://www.biomap-imi.eu/). M. R. Ardern-Jones has undertaken consultancy or received sponsorship or his department has received funding from Abbvie, Almirall, Ducentis, Janssen, Leo, Lilly Pfizer, and Sanofi Genzyme. S. J. Brown holds a Wellcome Trust Senior Research Fellowship (220875/Z/20/Z) and has received research funding but no personal payments from Pfizer, Abbvie, Janssen, Sosie-Heptares, and the European Lead Factory. J. R. Ingram receives a stipend as Editor-in-Chief of the *British Journal of Dermatology* and an authorship honorarium from UpToDate. He is a consultant for Boehringer Ingelheim, ChemoCentryx, Novartis, and UCB Pharma and has served on advisory boards for Insmed, Kymera Therapeutics, and Viela Bio, all in the field of hidradenitis suppurativa (HS). He is co-copyright holder of HiSQOL, Investigator Global Assessment and Patient Global Assessment instruments for HS. His department receives income from copyright of the Dermatology Life Quality Instrument (DLQI) and related instruments. A. D. Irvine received consulting fees from Arena, Amirall, Pfizer, Regenron, Sanofi, Novarti, Abbvie, Benevolent Ai, and Lilly; payment or honoraria for lectures, presentations, speakers bureaus, manuscript writing, or educational events from Leo, Abbvie, Lilly, and Sanofi; participation on a Data Safety Monitoring Board or Advisory Board for Novartis (paid); leadership or fiduciary role in other board, society, committee, or advocacy group at International Eczema Council (unpaid) and Irish Hospital Consultants Association (unpaid). G. Ogg is funded by the Medical Research Council and NIHR Oxford Biomedical Research Centre and has received research awards or undertaken advisory roles for Sanofi, Leo Pharma, Eli Lilly, UCB, Novartis, Janssen, and BMS. N. J. Reynolds has performed consultancy work/lectures for Almirall UK LTD, Abbvie, LEO Pharma, Lilly UK, and Novartis UK Sanofi Genzyme though Newcastle University. Income to Newcastle University, no personal income (over last 5 years). R. B. Warren has received research grants from AbbVie, Almirall, Amgen, Celgene, Janssen, Lilly, Leo, Medac, Novartis, Pfizer, and UCB and consulting fees from AbbVie, Almirall, Amgen, Arena, Astellas, Avillion, Biogen, Boehringer Ingelheim, Bristol Myers Squibb, Celgene, DiCE, GSK, Janssen, Lilly, Leo, Medac, Novartis, Pfizer, Sanofi, Sun Pharma, UCB, and UNION. R. T. Woolf is principal or co-investigator in clinical trials—Abbvie, Amgen, Anaptys Bio, Boehringer Ingelheim, Bristol Myers Squibb, Celgene, Eli Lilly, Galderma, Janssen-Cilag, Kymab, Leo Pharma, Pfizer, Sanofi, and UCB; received honoraria from and consultancy work for Abbvie, Eli Lilly, Janssen-Cilag, Leo Pharma, Novartis, Sandoz, Sanofi, and UCB; and received honoraria from NICE (clinical expert). P. I. Spuls has done consultancies in the past for Sanofi 111017 and AbbVie 041217 (unpaid), received a departmental independent research grants for TREAT NL registry from Pharma since December 2019, is involved in performing clinical trials with many pharmaceutical industries that manufacture drugs used for the treatment of, for example, psoriasis and atopic dermatitis, for which financial compensation is paid to the department/hospital and is chief investigator (CI) of the systemic and phototherapy atopic eczema registry (TREAT NL) for adults and children. C. Flohr is Chief Investigator of the UK National Institute for Health Research-funded TREAT (ISRCTN15837754) and SOFTER (Clinicaltrials.gov: NCT03270566) trials as well as the UK-Irish Atopic eczema Systemic Therapy Register (A-STAR; ISRCTN11210918) and a principle investigator in the European Union (EU) Horizon 2020-funded BIOMAP Consortium (http://www.biomap-imi.eu/). He also leads the EU Trans-Foods Consortium. His department has received funding from Sanofi-Genzyme for skin microbiome work. M. A. Middelkamp-Hup has done consultancies for Sanofi, Pfizer, and Leo Pharma and is one of the main investigators of the TREAT NL registry.
